# Supplementary material for: Antibiotics utilization patterns among tertiary care hospitals in Ethiopia
Source: PLoS One. 2025 Oct 27;20(10):e0335027. doi: 10.1371/journal.pone.0335027 (PMC12558456; doi:10.1371/journal.pone.0335027)
Supplement: Attachment — Submitted filename: Comment for the manuscript.docx [file pone.0335027.s002.docx]

|  | General comment | There are issues with English language writing in the paper  Overall, there is a casual approach. With three tables missing in the manuscript. And repetition of the same data in the tables provided. |
| --- | --- | --- |
|  | Introduction | The study has already been conducted. |
|  | Result | Table 2 is not clear. Need to rewrite. Under column “drugs,” the name of the drug needs to be mentioned.  Tables 3,5, and 6 are missing in the text  Explanation for low antibiotic consumption in ICU?  There is repetition of the data in the tables provided.  Earlier tables already mentioned the consumption of different antibiotics, however, antibiotics names are not mentioned there. It represents as drug 1,2,3, etc.  That is not a way of presentation in a scientific paper. Practically no need for table 7.  Which were the common CAI and HAI?  Inclusion criteria are not clear.  Antibiotics mentioned under ATC were included, or those in EML of the country were included?  If the patient was in hospital for a longer period, were all the antibiotics he received included?  If the patient developed HAI a few days after hospital admission, which diagnosis was included? Diagnosis at the time or entry of current diagnosis? |
