# Supplementary material for: Antibiotics utilization patterns among tertiary care hospitals in Ethiopia
Source: PLoS One. 2025 Oct 27;20(10):e0335027. doi: 10.1371/journal.pone.0335027 (PMC12558456; doi:10.1371/journal.pone.0335027)
Supplement: Attachment — Submitted filename: Response to reviewers.docx [file pone.0335027.s003.docx]

**Response to reviewers**

1. **Additional Editor Comments:**

Comment 1: The paper requires intensive revision.

Response 1: We thoroughly reviewed the manuscript, and a major revision was done

Comment 2: Avoid words like 'see figure 1' etc. It is better written as (Figure 1)

Response 2: Avoided

Comment 3: Follow the PLOS ONE manuscript writing protocol.

Response 3: We followed the PLOS ONE manuscript writing guidelines during manuscript preparation

2. **Reviewers comments**

**Comments 1:**  Is the manuscripts technically sound, and do the data support the conclusions?

The manuscript must describe a technically sound piece of scientific research with data that supports the conclusions. Experiments must have been conducted rigorously, with appropriate controls, replication, and sample sizes. The conclusions must be drawn appropriately based on the data presented.

Reviewer #1: Partly

Reviewer #2: Partly

**Response 1:** Yes, the manuscript is technically sound, and the data support the conclusion. The issue of technical soundness was well mentioned in the methods and materials part of the study on pages 5-8. The data also support the conclusion mentioned. There was a high consumption of antibiotics in tertiary hospitals in Ethiopia, and three-fourths of antibiotics were prescribed from the "Watch" group, which was the inverse of the WHO recommendation. This was mentioned in tables 2 and 8 of the manuscript on pages 12 and 16.

**Comment 2:**  Has the statistical analysis been performed appropriately and rigorously?

Reviewer #1: Yes

Reviewer #2: Yes

**Response 2:** Sure, the appropriate statistical analysis for each specific objective was performed by using SPSS version 21 and Microsoft Excel.

**Comment 3:** Have the authors made all data underlying the findings in their manuscript fully available?

The [PLOS Data policy](http://www.plosone.org/static/policies.action#sharing) requires authors to make all data underlying the findings described in their manuscript fully available without restriction, with rare exceptions (please refer to the Data Availability Statement in the manuscript PDF file). The data should be provided as part of the manuscript or its supporting information, or deposited in a public repository. For example, in addition to summary statistics, the data points behind means, medians, and variance measures should be available. If there are restrictions on publicly sharing data—e.g., participant privacy or use of data from a third party—those must be specified.

Reviewer #1: Yes

Reviewer #2: No

**Response 3:** All data underlying the findings were included in the manuscript and are fully available

**Comment 4:** Is the manuscript presented in an intelligible fashion and written in standard English?

PLOS ONE does not copyedit accepted manuscripts, so the language in submitted articles must be clear, correct, and unambiguous. Any typographical or grammatical errors should be corrected at revision, so please note any specific errors here.

Reviewer #1: Yes

Reviewer #2: No

**Response 4:** Sure, the manuscript was presented in an intelligible fashion and written in standard English. We also exhaustively reviewed and edited the whole parts of the manuscript.

**Comment 5:** Timeliness of Data:

The study was conducted three years ago, and antibiotic prescribing practices or resistance patterns may have since evolved. The authors should address how this temporal gap might affect the current relevance of their findings.

Reviewer #1: Yes

**Response 5:** Since this is a retrospective study, we believe it can provide benchmarking data for antibiotics utilization patterns for study tertiary care hospitals and all other tertiary care hospitals in Ethiopia for further prospective studies that have a recent temporal relevance. The result can also, provide an evidence for antimicrobial prescribing practice and stewardship in study setting.

**Comment 6:** Ethical Approval/Consideration

While retrospective studies often involve anonymized data, the manuscript should explicitly state whether ethical approval (e.g., IRB reference number) was obtained to ensure compliance with research integrity standards.

3. Study Design Limitations:

o The retrospective cross-sectional design precludes causal inferences (e.g., linking prescribing patterns directly to resistance rates).

o Excluding outpatient and pediatric data omits significant antibiotic use contexts. Recommendation: Future studies should expand methodology to include these populations and integrate local antibiograms to correlate prescribing with resistance trends.

Reviewer #1: Yes

**Response 6:** The ethical approval was obtained as mentioned on page number 9, and the latter uploaded separately as IRB.

Regarding a limitation of the study design, it a valid comments, we included the recommendation on manuscript page number 20 in the conclusion part.

**Comment 7:** Generalizability Concerns:

o Findings from five public tertiary hospitals may not reflect practices in primary/secondary care or private sectors.

o Regional variability (4 regions) may not capture national heterogeneity. Recommendation: Acknowledge this limitation and suggest multi-center studies for broader applicability.

Reviewer #1: Yes

**Response 7:** Valid recommendation and included in the limitation and conclusion parts, page 20

**Comment 8:** Methodological Gaps:

o The DDD metric does not account for dosing adjustments (e.g., renal impairment) or pediatric use.

o Clinical outcomes (e.g., treatment success, resistance emergence) are not analyzed. Recommendation: Discuss these limitations and propose linking future data to patient outcomes.

Reviewer #1: Yes

**Response 8:** Methodological Gaps: Valid recommendation and included in the limitation and conclusion parts, on page 20

Regarding clinical outcomes, we really appreciate your view; however, it is not the objective of the current study, but we had considered it in another manuscript.

**Comment 9:** Presentation Issues:

o Tables 2–4 contain repetitive data and could be consolidated for clarity.

o A visual summary (e.g., a bar chart of AWaRe group deviations from WHO targets) would improve readability.

Reviewer #1: Yes

**Response 9:** We appreciate your view; however, Table No, 2-4 show different data.

Table 2: shows the total number of drugs prescribed and the ratio of antibiotics from prescribed drugs.

Table 3: shows the number of antibiotics prescribed per patient and the antibiotic exposure rate.

Table 4: shows the amount of antibiotics consumed based on the indication for antibiotic use.

**Comment 10:** Discussion Enhancements:

o Contextualize findings with regional AMR surveillance data (e.g., resistance rates for ceftriaxone).

o Compare results with Ethiopia’s AMR National Action Plan (if available) and propose specific stewardship interventions (e.g., audit/feedback, surgical prophylaxis guidelines).

Reviewer #1: Yes

**Response 10:** Valid comments, included in revised manuscript of the conclusion part, page 25

**Comment 11:** Policy and Recommendations:

o Detail actionable strategies (e.g., formulary restrictions, prescriber education).

o Include cost-analysis of antibiotic misuse (e.g., budget impact of Watch-group overuse).

o Advocate for hospital-specific formularies prioritizing "Access" group antibiotics and training on de-escalation protocols.

Reviewer #1: Yes

**Response 11:** Policy recommendation was included, on discussion part, page 27. However, we haven’t considered the cost analysis of antibiotic misuse, because it was beyond our objective.

**Comment 12:** Needs to work on the English language presentation

Reviewer #2

**Response 12:** The language issue was thoroughly reviewed and edited

**Comment 13:** Tables No. 3, 5, and 6 are missing in the manuscript

Reviewer #2

**Response 13:** We appreciate your vigilant view; we had uploaded table No. 3, 5, and 6 separately categorized as others due to the large size of the tables in first manuscript. Now, all tables were included in revised manuscript, pages 13,15, and 17

**Comment 14:** Table no 2 mentions Drugs with drug names are mentioned as 1,2,3,4,5. This does not explain anything.

Reviewer #2

**Response 14:** Drug 1, Drug 2, Drug 3, Drug 4, Drug 5, Drug 6 and Drug 7 indicate types of medications prescribed for patients as 1^st^ ,2^nd^ ,3^rd^ ,4^th^ ,5^th^ ,6^th^ and 7^th^ round during patients’ hospital stay. Included in revised manuscript, page number 12.

**Comment 15:** What is the novelty of the data in the study? The study was already conducted.

Reviewer #2

**Response 15:** The rationale and nobility of the current study were, despite limited studies on utilization patterns of antibiotics in Ethiopia, most research has been conducted at single centers, and data on antibiotic utilization patterns across multiple hospitals remain scarce. Page 5
