# Supplementary material for: Antibiotics utilization patterns among tertiary care hospitals in Ethiopia
Source: PLoS One. 2025 Oct 27;20(10):e0335027. doi: 10.1371/journal.pone.0335027 (PMC12558456; doi:10.1371/journal.pone.0335027)
Supplement: Attachment — Submitted filename: Response_to_reviewers_auresp_2.docx [file pone.0335027.s004.docx]

**Response to reviewers**

1. **Additional Editor Comments:**

Comment 1: It is better the author order, ‘Materials and methods.

Response 1: edited on page #5

Comment 2: The figure 1, description should be placed within the manuscript where cited below it.

Response 2: included

Comment 3: Proofread is also required.

Response 3: checked many times for spelling, fellow and coherence issues

2. **Reviewers comments**

**Comment 1:**  Inclusion criteria are vague and lack specification. Antibiotics outside ELM were not included?
Inclusion criteria mentions “Antibiotics prescribed for initial diagnosis were included”. Please clarify.

Reviewer #2: Yes

**Response 1:** Valid comments, inclusion criteria were clearly stated on page #5

**Comment 2:** Ethics permission from the registered ethics committee of each hospital participating in the study is required to collect data. Verbal permission may not be sufficient or authentic.

Reviewer #2: Yes

**Response 2:** The copy of ethical clearance obtained from the institutional Ethical Review Board (IRB) of Jimma University, Institute of Health and the official support letter submitted to each study tertiary care hospitals, the hospital administration bodies linkage to specific department in which study conduct through inter-facility memo and call. Then data collection process was started as mentioned in ethical issues on page # 8. Hence, specific ethical clearance from each hospital was not required during our data collection period.

**Comment 3:** The study design is mentioned as “retrospective cross-sectional study”; however, the exact time point of data collection is not mentioned in the manuscript. Only the duration during which the data was assessed, as has been mentioned

Reviewer #2: Yes

**Response 3:** The exact time point of data collection was April 17/2024 to July 03/2024 stated on page # 9

**Comment 4:** The number of female participants in the text and table is different.

Reviewer #2: Yes

**Response 4:** edited on page #11

**Comment 5:** The legend for Table 2 is not clear. What are 1,2,3,4,5,6,7, rounds of prescribing? Table 4: column 1st: What does drug 1,2,3,4,56,7 indicate?.

Reviewer #2: Yes

**Response 5:** valid comments, edited and explained on page #12

Table 2: Total number of drugs prescribed and number and ratio of antibiotics from total prescribed drugs.

Drug 1, Drug 2, Drug 3, Drug 4, Drug 5, Drug 6 and Drug 7 were prescribed medication lists written/prescribed as 1^st^ ,2^nd^ ,3^rd^ ,4^th^ ,5^th^ ,6^th^ and 7^th^ round(times) during patients’ hospital stay

**Comment 6:** There are multiple instances of spelling errors. (e.g., a bar chart of AWaRe group deviations from WHO targets) would improve readability.

Reviewer #2: Yes

**Response 6:** exhaustively checked
